# *Yersinia enterocolitica*: Epidemiological Studies and Outbreaks

**DOI:** 10.4061/2011/239391

**Published:** 2011-10-16

**Authors:** Atiqur Rahman, Tania S. Bonny, Siriporn Stonsaovapak, Chiraporn Ananchaipattana

**Affiliations:** ^1^Department of Microbiology, University of Dhaka, Dhaka 1000, Bangladesh; ^2^Applied Microbiology Department, Institute of Food Research and Product Development (IFRPD), Kasetsart University, Bangkok 10903, Thailand; ^3^Department of Biology, Faculty of Science and Technology, Rajamangala University of Technology Thanyaburi, Panthumthani 121100, Thailand

## Abstract

*Yersinia enterocolitica* is the most common bacteriological cause of gastrointestinal disease in many developed and developing countries. Although contaminated food is the main source of human infection due to *Y. enterocolitica*, animal reservoir and contaminated environment are also considered as other possible infection sources for human in epidemiological studies. Molecular based epidemiological studies are found to be more efficient in investigating the occurrence of human pathogenic *Y. enterocolitica* in natural samples, in addition to conventional culture based studies.

## 1. Introduction

Foodborne diseases are a widespread and growing public health problem in developed and developing countries [1]. Amongst those, yersiniosis due to infection with the bacterium *Yersinia enterocolitica* is the frequently reported zoonotic gastrointestinal disease after campylobacteriosis and salmonellosis in many developed countries, especially in temperate zones [2]. Within developed countries, incidences of yersiniosis and foodborne outbreaks are appeared to be lower in the United States than many European countries [3–5]. In European countries, numbers of reported cases of human in England and Wales are lower than those in other European countries where fewer than 0.1 cases of yersiniosis per 100,000 individuals were reported in the United Kingdom in 2005, in contrast to 12.2 in Finland and 6.8 in Germany [6]. On the other hand, the high prevalence of gastrointestinal illness including fatal cases due to yersiniosis is also observed in many developing countries like Bangladesh [7], Iraq [8], Iran [9], and Nigeria [10], which indicates major underlying food safety problems in low- and middle-income countries. Worldwide, infection with *Y. enterocolitica* occurs most often in infants and young children with common symptoms like fever, abdominal pain, and diarrhea, which is often bloody. Older children and young adults are not out of risk. The predominant symptoms within these age groups are right-sided abdominal pain and fever, sometimes confused with appendicitis. Occasionally, the *Y. enterocolitica* associated complications such as skin rash, joint pains, or spread of bacteria to the bloodstream can also occur. 

Although *Y. enterocolitica* is a ubiquitous microorganism, the majority of isolates recovered from asymptomatic carriers, infected animals, contaminated food, untreated water, and contaminated environmental samples are nonpathogenic having no clinical importance [11]. At the same time, the epidemiology of *Y. enterocolitica* infections is complex and remains poorly understood because most sporadically occurred cases of yersiniosis are reported without an apparent source [3, 12–14]. However, most pathogenic *Y. enterocolitica* strains associated with human yersiniosis belong to bioserotypes 1B/O:8, 2/O:5,27, 2/O:9, 3/O:3, and 4/O:3. Within these reported strains, fully pathogenic strains carry an approximately 70 kb plasmid termed pYV (plasmid for *Yersinia *virulence) [15] that encodes various virulence genes (*tccC*, *yadA*, *virF*, *ysa)* with traditional chromosomal virulence genes (*inv*, *ail*, *yst*) whereas other pathogenic strains, having no pYV plasmid, produce a thermostable enterotoxin (*yst*A) [16–18]. These virulence genes located in chromosome or plasmid of pathogenic* Y. enterocolitica *has been widely used to identify pathogenic strains in epidemiological studies for example, chromosomal *ail* gene [19, 20].

## 2. Epidemiological Studies and Outbreaks

Many factors related to the epidemiology of *Y. enterocolitica,* such as human and nonhuman sources, and contamination routes in foods remain obscure in developing countries and tropical regions of developed countries. Additionally, epidemiological data on the prevalence of pathogenic* Y. enterocolitica *in animals in developed countries are missing as the reporting of this pathogen in animals is not mandatory in most European countries [26].

### 2.1. Animal Reservoirs Involved in Zoonosis

**Figure 1:**
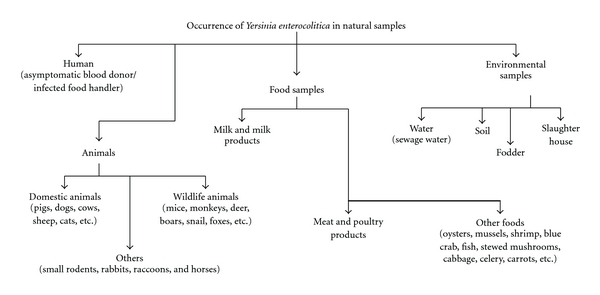
Occurrence of *Y. enterocolitica *in natural samples.

Animals have long been suspected of being significant reservoirs for *Y. enterocolitica* and, therefore, sources of human infections [3]. Numerous studies have been carried out to isolate *Y. enterocolitica* strains from a variety of animals (Figure 1) [56]. Interestingly, most of the strains isolated from the animal kingdom carry unique serotypes of *Y. enterocolitica *compared to the strains isolated from humans with yersiniosis.

Pigs have been shown to be a major reservoir of pathogenic *Y. enterocolitica *involved in human infections, particularly for strains of bioserotype 4/O:3 which has been almost exclusively isolated in European countries like Denmark, Italy, Belgium, Spain, and Sweden [24, 64]. The rate of isolation of *Y. enterocolitica* including bioserotype 4/O:3 from tonsils and tongues of pigs is generally greater than the rate of isolation from cecal or fecal materials [20].

Occasionally, pathogenic *Y. enterocolitica* strains, mostly of bioserotype 4/O:3, have also been isolated from dogs and cats [82]. Although pigs are the primary source of human infection with *Y. enterocolitica* throughout world, these pets may also be a potential source of human infection with pathogenic *Y. enterocolitica *because of their intimate contact with people, especially young children [28].

In addition with mostly isolated bioserotype 4/O:3, *Y. enterocolitica* strains of biotypes 2 and 3 and serotypes O:5,27, O:8, and O:9 have also been isolated from slaughter pigs, cows, sheep, and goats; however, the reservoir of these bioserotypes is not clearly established [81, 83–85]. In above cases, contamination of pluck sets (tongue, tonsils, and trachea hanging together with thoracic organs such as lungs, liver, and heart) and carcasses with enteropathogenic *Yersinia* from tonsils and feces may occur during the slaughtering stage [5, 82, 86–88]. On the other hand, strains of very rare bioserotypes, such as bioserotype 5/O:2,3, have been isolated from sheep, hares, and goats and bioserotype 3/O:1,2a,3 from chinchillas (small rodent). Thus, the patterns of the pathogenic strains isolated from humans with yersiniosis compared to those from the animals suggest that the human infection due to *Y. enterocolitica* originated from the animals.

### 2.2. Contaminated Food Involved in Infections

**Figure 2:**
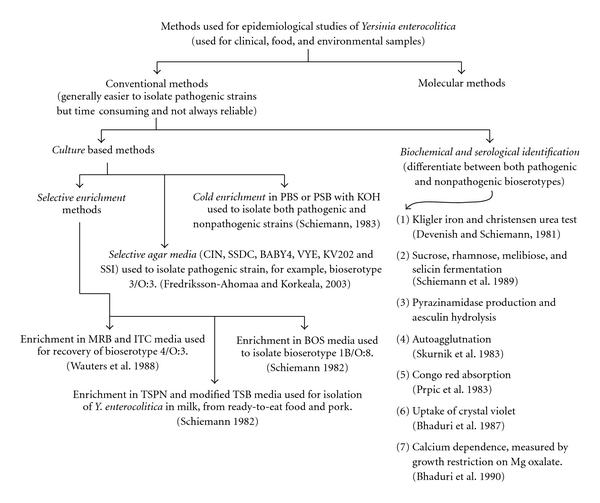
Methods used for epidemiological studies of *Y. enterocolitica*-1. Selective enrichment methods [43]; selective agar media [11]; cold enrichment method [57]; biochemical & serological identification methods [58–63]. (PBS: Phosphate buffered saline; PSB: Phosphate-buffered saline with sorbitol and bile salts; MRB: Modified Rappaport broth containing magnesium chloride, malachite green, and carbenicillin; ITC: Modified Rappaport base supplemented with irgasan, ticarcillin, and potassium chlorate; BOS: Bile-oxalate-sorbose medium; TSB: Tryptic soy broth; TSPN: TSB with polymyxin and novobiocin; CIN: Cefsulodin-irgasan-novobiocin; SSDC: *Salmonella-Shigella* deoxycolate calcium chloride; VYE: Virulent *Yersinia enterocolitica*; SSI: Statens Serum Institute, Copenhagen, Denmark, enteric medium).

**Figure 3:**
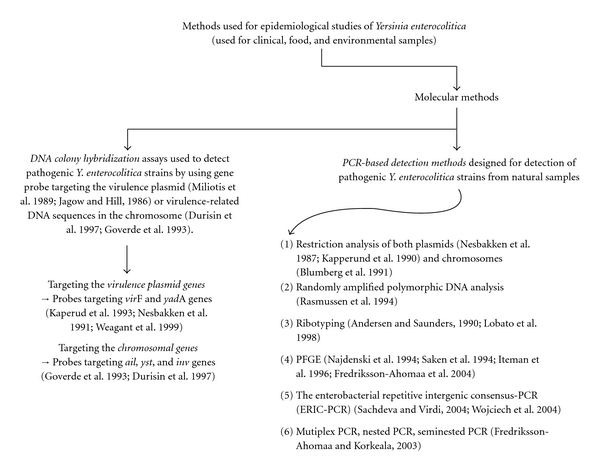
Methods used for epidemiological studies of *Y. enterocolitica*-2. DNA colony hybridization assays [51, 65–70]; PCR based detection methods [11, 71–81]. (*inv*: gene for invasin, an outer membrane protein that is required for efficient translocation of bacteria across the intestinal epithelium; *ail*: gene for adhesin, an outer membrane protein that may contribute to adhesion, invasion and resistance to complement-mediated lysis; *yst*: gene for heat-stable enterotoxin that may contribute to the pathogenesis of diarrhea associated with acute yersiniosis; *virF*: gene for transcriptional activator; *yadA*, gene for *Yersinia* adhesin A; PFGE: pulsed field gel electrophoresis).

Food has been proposed to be the main source of intestinal yersiniosis although pathogenic isolates have seldom been recovered from food samples [105]. The low recovery rates of pathogenic *Y. enterocolitica* in food samples may be due to limited sensitivity of culture methods [11]. However*, Y. enterocolitica* has been isolated from milk and milk products, egg products, raw meats (beef, pork, and lamb) and poultry, vegetables, and miscellaneous prepared food products. The occurrence of pathogenic *Y. enterocolitica* in natural sample including foods has been estimated by both culture- and molecular-based methods (Table 1, Figures 2 and 3).

#### 2.2.1. Contaminated Meat and Poultry Products Correlated with yersiniosis

Indirect evidence considering food, particularly pork and pork products, indicates that there is an important link between consumption of raw, undercooked, or improperly handled pork product and human *Y. enterocolitica* infections [20]. This positive correlation between the consumption of raw or undercooked pork and the prevalence of yersiniosis has been demonstrated in case-control studies [32, 64, 106–109]. Using molecular techniques, *ail*-positive *Y. enterocolitica* strains were detected in raw pork samples (loin, fillet, chop, ham, and minced meat) and in ready-to-eat pork products [31]. However, the isolation rates of pathogenic bioserotypes of *Y. enterocolitica *have been low in raw pork, except for in edible pig offal, with the most common type isolated being bioserotype 4/O:3 (Table 2). In other studies, pathogenic *yst*-positive *Y. enterocolitica* strains have been isolated from ground beef [29] but not detected in chicken food samples [110].

#### 2.2.2. Contaminated Milk and Milk Products Associated with Human Disease

*Y. enterocolitica* has been isolated from raw milk in many countries, like Australia, Canada, Czechoslovakia, and USA. There were also a few reports on the isolation of this pathogenic strain associated with human disease from pasteurized milk [4, 111]. It may be due to the malfunction in the pasteurization process leading to inadequate treatment or postprocess contamination, or it may be due to the contamination with heat-resistant strains of *Y. enterocolitica*. So, the presence of this pathogen in pasteurized milk should be a cause for concern. However, heat-resistant strains of *Y. enterocolitica* have not been still reported in milk samples.

#### 2.2.3. Other Contaminated Foods Involved in Outbreaks

Strains of *Y. enterocolitica* have been isolated from oysters, mussels, shrimp, blue crab, fish, salad, stewed mushrooms, cabbage, celery, and carrots [112]. In Korea, Lee et al. [113] isolated *ail*-positive *Y. enterocolitica* strain of bioserotype 3/O:3 from ready-to-eat vegetables, which indicate that vegetables can be a source of human infection. Furthermore, Sakai et al. [34] reported an outbreak of food poisoning by *Y. enterocolitica* serotype O:8 in Japan where salad was proposed the cause of infection. Recently, *Y. enterocolitica* 2/O:9 has been isolated from chicken eggshell surfaces in Argentina [114]. Contamination of the egg surface might have occurred from contact with other *Y. enterocolitica*-contaminated animal products, such as pork product, during collection on farms or during transportation or handling in retail shops.

### 2.3. Contaminated Environment Reported as Source of Infection

Most of the *Y. enterocolitica* isolates recovered from environmental samples, including the slaughterhouse, fodder, soil, and water, have been nonpathogenic [89, 115–119]. Occasionally, strains of bioserotype 4/O:3 have been isolated from the slaughterhouse [120, 121] and sewage water [50]. Within the environmental sampling sites, drinking water has been relatively widely investigated and revealed to be a significant reservoir for nonpathogenic *Y. enterocolitica. *However, Sandery et al. [35] detected pathogenic *Y. enterocolitica* in environmental water by molecular studies. In a case-control study, untreated drinking water has been reported to be a risk factor for sporadic *Y. enterocolitica* infections in Norway [107]. Recently, Falcão et al. [122] tested 67 *Y. enterocolitica* strains isolated in Brazil from untreated water for the presence of virulence genes. They found that all 38 strains of serotype O:5,27 possessed *inv, ail*, and *ys*t genes, suggesting that untreated water may be responsible for the human infection with *Y. enterocolitica*. In another study, *Y. enterocolitica* O:8 strains have been isolated from stream water in Japan, which indicate that stream water may be a possible infection source for human *Y. enterocolitica* O:8 infections [84, 123].

## 3. Conclusion

Epidemiological studies of human infection with *Y. enterocolitica *(Table 3) constitute an important element in the exploitation of apparent sources and contamination routes of human yersiniosis and in the development and implementation of effective control strategies to prevent future outbreaks. Efficient laboratory methods used for epidemiological study are also a vital requirement in *Y. enterocolitica's *monitoring and control purposes. Molecular methods should be needed with conventional culture methods to provide a better estimation of epidemiology of *Y. enterocolitica *particularly pathogenic strains in natural samples

## Figures and Tables

**Table 1:** Detection of pathogenic *Y. enterocolitica* in natural samples with PCR and culture methods.

Sample	No. of samples	No. of culture^+ve^samples^a^ (%)		No. of PCR^+ve^samples (%)		References
*Animal*						
Pig tonsils	185	48	(26)	58	(31)	Fredriksson-Ahomaa et al. [21]
	252	0		90	(36)	Boyapalle et al. [22]
	24	15	(63)	18	(75)	Nesbakken et al. [23]
	829	411	(50)	0		Martínez et al. [24]
	630	278	(44)	0		Martínez et al. [25]
	212	72	(34)	186	(88)	Fredriksson-Ahomaa et al. [26]
Pig faeces	255	0		80	(31)	Boyapalle et al. [22]
	24	3	(13)	3	(13)	Nesbakken et al. [23]
	2793	114	(4)	345	(12)	Bhaduri et al. [27]
	150	3	(2)	0		Okwori et al. [10]
Mesenteric l. n.	257	0		103	(40)	Boyapalle et al. [22]
	24	1	(4)	2	(8)	Nesbakken et al. [23]
Submaxillary l. n.	24	1	(4)	3	(13)	Fredriksson-Ahomaa et al. [20]
Sheep feces	200	2	(1)	0		Okwori et al. [10]
Dog feces	448	0		6	(1)	Wang et al. [28]
*Food* ^ b^						
Pig tongues	15	7	(47)	10	(67)	Vishnubhatla et al. [29]
	99	79	(80)	82	(83)	Fredriksson-Ahomaa and Korkeala [11]
Pig offal^c^	110	38	(35)	77	(70)	Fredriksson-Ahomaa et al. [20]
Chitterlings	350	8	(2)	278	(79)	Boyapalle et al. [22]
Ground pork	350	0		133	(38)	Fredriksson-Ahomaa et al. [20]
	100	32	(32)	47	(47)	Vishnubhatla et al. [29]
Ground beef	100	23	(23)	31	(31)	Fredriksson-Ahomaa et al. [20]
Minced pork	255	4	(2)	63	(25)	Fredriksson-Ahomaa and Korkeala [11]
Pork^d^	300	6	(2)	50	(17)	Johannessen et al. [30]
	91	6	(7)	9	(10)	Lambertz & Danielsson-Tham [31]
	62	0		20	(32)	Grahek-Ogden et al. [32]
Chicken	43	0		0		Fredriksson-Ahomaa et al. [11]
Fish	150	0		0		Okwori et al. [10]
Heated soup	100	3	(3)			Okwori et al. [10]
Cow milk	250	3	(1)			Okwori et al. [10]
Lettuce	250	0		3	(3)	Okwori et al. [10]
Tofu	50	0		6	(12)	Vishnubhatla et al. [29]
Vegetables	27	1	(4)	4	(15)	Cocolin & Comi [33]
Salad	42	16	(38)	16	(38)	Sakai et al. [34]
*Environment*						
Water	105	1	(1)	11	(10)	Sandery et al. [35]
Slaughterhouse/ Farm	89	5	(6)	12	(13)	Fredriksson-Ahomaa et al. [36]
	46	44	(96)	0		Martínez et al. [24]
	45	31	(61)	0		Martínez et al. [25]

^a^Pathogenicity of isolates confirmed, ^b^all meat samples are raw, ^c^liver, heart, kidney, ^d^except pig offal & tongues, and ^+ve^positive.

**Table 2:** Detection of pathogenic *Y. enterocolitica* in pork products by culture methods (partially adapted from Fredriksson-and Korkeala [11]).

Sample	No. of samples	No. of samples positive for				Country of origin of sample	Reference
		O:3	O:5,27	O:8	O:9		
Tongue	302	165			3	Belgium	Wauters [37]
	37	11				Canada	Schiemann [38]
	31	2		6		USA	Doyle et al. [39]
	47	26				Norway	Nesbakken [40]
	50	20				Japan	Shiozawa et al. [41]
	125	8				Spain	Ferrer et al. [42]
	29	28				Belgium	Wauters et al. [43]
	40	6			2	The Netherlands	de Boer and Nouws [44]
	55	14				Germany	Karib and Seeger [45]
	86	2				Italy	de Guisti et al. [46]
	99	79				Finland	Fredriksson-Ahomaa et al. [47]
	20	15				Germany	Fredriksson-Ahomaa et al. [48]

Tonsil	89	81			8	Belgium	Martínez et al. [24]
	137	136	1			Italy	Martínez et al. [24]
	185	185				Spain	Martínez et al. [24]
	212	69	6		1	Switzerland	Fredriksson-Ahomaa et al. [26]

Offal^a^	34	17				Finland	Fredriksson-Ahomaa et al. [36]
	16	5				Finland	Fredriksson-Ahomaa et al. [47]
	100	46				Germany	Fredriksson-Ahomaa et al. [48]

Pork^b^	91	1		1		Canada	Schiemann [38]
	127	1				Norway	Nesbakken et al. [49]
	70	22			3	Japan	Shiozawa et al. [41]
	267	6				Denmark	Christensen [50]
	50	12				Belgium	Wauters et al. [43]
	400	3			1	The Netherlands	de Boer and Nouws [44]
	45	8				Norway	Nesbakken et al. [51]
	67	1	8^c^	3		China	Tsai and Chen [52]
	48	1			1	Germany	Karib and Seeger [45]
	40	2	4		1	Ireland	Logue et al. [53]
	1278	64	14			Japan	Fukushima et al. [54]
	255	4				Finland	Fredriksson-Ahomaa et al. [55]
	300	6				Norway	Johannessen et al. [30]
	120	14				Germany	Fredriksson-Ahomaa et al. [36]
	60				20	Norway	Grahek-Ogden et al. [32]

^a^Offal, excluding tongue, ^b^other pork products, excluding offal, ^c^isolates belonging to serotype O:5 and showing autoagglutination activity and calcium-dependent growth.

**Table 3:** Epidemiological studies of human infection with *Y. enterocolitica*.

Year	Country	Outcome of the study	References
1981–1990	Georgia	Report of 84 clinical isolates of *Y. enterocolitica*, the most frequently reported serotypes were O:5; O:10,46; O:6,30	Sulakvelidze et al. [89]
1982–1991	The Netherlands	Analysis of clinical information from 261 Dutch patients with gastrointestinal infections caused by *Y. enterocolitica* serotypes O:3 and O:9	Stolk-Engelaar and Hoogkamp-Korstanje [90]
1982^a^	Canada	Outbreak of gastroenteritis among hospitalized patients associated with *Y. enterocolitica* serotype O:5	Ratnam et al. [91]
1982–1985	Canada	Examination of 125 isolates of *Y. enterocolitica*, serotypes O:7,8; O:5; O:6,30, were frequently obtained from symptomatic patients	Noble et al. [92]
1983	Finland	Report of 46 fecal isolates of *Y. enterocolitica*, including two serotypes O:7; O:6, associated with occurrence	Skurnik et al. [60]
1984^a^	Bangladesh	Case report of a fatal diarrheal illness associated with serotypes O:7; O:8	Butler et al. [7]
1984^a^	Hong Kong	Report of *Y. enterocolitica*-associated septicemia in four patients regarding serotypes O:17	Seto and Lau [93]
1984-1985	UK	Report of two nosocomial outbreaks of *Y. enterocolitica* serotypes O:10; O:6 infections in hospitalized children	Greenwood and Hooper [94]
1986^a^	UK	Case report of nosocomial transmission of serotypes O:6,30 associated with gastroenteritis	McIntyre and Nnochiri [95]
1986–1992	Canada	Report of 79 symptomatic children with culture-proven infection, including serotypes O:5; O:6,30; O:7,8	Cimolai et al. [96]
1987	UK	Report of 77 *Y. enterocolitica* strains from patients, including serotypes O:6,30; O:7	Greenwood and Hooper [97]
1987-1988	Australia	Report of 11 cases of *Y. enterocolitica* enteritis, including most frequently serotypes O:6,30	Butt et al. [98]
1987–1989	Chile	A prospective case-control study of infants with diarrhoea in Chile, showing a significantly reported serotypes O:6; O:7,8; O:7; O:10	Morris et al. [99]
1988–1991	Nigeria	Of nine strains of *Y. enterocolitica* obtained from stool samples of children with diarrhoea	Onyemelukwe [100]
1988–1993	New Zealand	Of 918 isolates of *Y. enterocolitica* from symptomatic patients	Fenwick and McCarthy [101]
1968–2000	Brazil	Of 106 strains (selected from the collection of the Yersinia Reference Laboratory in Brazil), 71 were bioserotype 4/O:3, isolated from human and animal clinical material, and 35 were of biotype 1A or 2, isolated from food	Falcão et al. [102]
2002	Iran	Report of 8 cases of *Y. enterocolitica* infection out of 300 children with acute diarrhoea aged 0–12 years who were attending a pediatric hospital in Tehran	Soltan-Dallal and Moezardalan [9]
2002–2004	Nigeria	Detection of *Y. enterocolitica *belonging to bioserotype 2/O:9 in investigating 500 human samples	Okwori et al. [10]
2004	Japan	Report of 16 cases food poisoning due to *Y. enterocolitica* serotype O:8	Sakai et al. [34]
2005–2006	Norway	Investigation of an outbreak involving 11 persons infected with *Yersinia enterocolitica* O:9	Grahek-Ogden et al. [32]
2001-2008	Germany	Almost 90% of *Y. enterocolitica* strains were diagnosed as serotype O:3	Rosner et al. [103]
2009^a^	Iraq	Identification of three children with diarrhoea caused by *Y. enterocolitica* infection	Kanan and Abdulla [8]
2009	Australia	Report of 1 outbreak with 3 cases due to consumption of roast pork contaminated with* Y. enterocolitica *	OzFoodNet sites [104]

^a^Year of publication.
